# Case report: A rare case of immunotherapy induced isolated left CN VI palsy in a patient with unresectable melanoma

**DOI:** 10.3389/fonc.2024.1330271

**Published:** 2024-02-12

**Authors:** Samantha Su Ping Low, Karim El-Shakankery, Ewan Brown, Alan Christie, Sally McCormack, Mark Stares

**Affiliations:** ^1^ Edinburgh Cancer Centre, Western General Hospital, NHS Lothian, Edinburgh, United Kingdom; ^2^ Dermatology, NHS Fife, Kirkcaldy, United Kingdom; ^3^ Edinburgh Cancer Research Centre, University of Edinburgh, Edinburgh, United Kingdom

**Keywords:** case report, immunotherapy, immune-related adverse events, cranial nerve palsy, unresectable melanoma

## Abstract

**Introduction:**

Immune checkpoint inhibitors are the mainstay of treatment in patients with unresectable or metastatic melanoma. Combination immunotherapy with ipilimumab and nivolumab has shown to improve survival outcomes as compared to single agent immunotherapy in these patients. Neurological immune-related adverse effects (irAEs) are uncommon and cranial nerve palsies are seen even more infrequently.

**Case presentation:**

A 66-year-old woman with a background of metastatic, unresectable melanoma with supraclavicular and axillary lymph nodal involvement presented with a headache, photophobia and diplopia 3 weeks after her first cycle of ipilimumab and nivolumab. She was subsequently diagnosed with a left-sided cranial nerve VI palsy and treated with high dose oral steroids and steroid eye drops, with complete resolution of symptoms. She also experienced Grade 3 dermatitis requiring topical steroids, Grade 2 hypothyroidism and vitiligo. She continues to have an excellent clinical and radiological response, despite further immunotherapy being suspended.

**Conclusion:**

This is the first reported UK case of immunotherapy-induced isolated cranial nerve VI palsy. Multiple irAEs are more common with combination immunotherapy and its occurrence is associated with more favourable outcomes in melanoma. Immunotherapy continues to revolutionise oncological care, but clinicians must be cognizant of unpredictable irAEs, which may require prompt assessment and intervention.

## 1 Introduction

Although malignant melanoma contributes to just 10% of all skin cancers, it is considerably more aggressive than other cutaneous malignancies, with a propensity to metastasise quickly (1, 2). Immune checkpoint inhibitors (ICI) are the mainstay of treatment in patients with unresectable stage III and stage IV melanoma. Such immune-mediated therapies have revolutionised the treatment of a cancer poorly responsive to conventional chemotherapies (2, 3). The multi-centre, multi-national CheckMate 067 trial showed that combination ipilimumab and nivolumab immunotherapies led to a remarkable median overall survival rate with durable treatment responses. These benefits were observed in both programmed cell death-ligand 1 (PD-L1) positive and negative tumours (4, 5).

Ipilimumab and nivolumab potentiate anti-tumour T-cell responses through cytotoxic T-lymphocyte antigen-4 (CTLA-4) and programmed cell death-1 (PD-1) receptor inhibition respectively (6, 7). However, inhibition of such checkpoints can inadvertently lead to a wide spectrum of unique immune-related adverse effects (irAEs). IrAEs are common, but unpredictable; they may occur at any time during the patient’s treatment course and can develop in almost any tissue type. Common sites for toxicity include the skin, gastrointestinal tract, liver and endocrine organs (8). Although combination immunotherapies increase tumour responses, they also increase the likelihood of irAEs when compared to ICI monotherapy; over 50% of CheckMate 067 participants treated with combination ipilimumab and nivolumab experienced Common Terminology Criteria for Adverse Events (CTCAE) Grade 3-4 events, compared to around 25% of participants with ipilimumab or nivolumab monotherapies (4, 9). Though most toxicities are reversible with glucocorticoid therapies, some are irreversible and/or result in chronic organ impairment (10).

Despite the above, neurological irAEs are relatively uncommon, accounting for approximately 2.8% of irAEs and are diverse in clinical presentation (11). The incidence of neurological irAEs is higher in the combined use of ipilimumab and nivolumab, compared to single-agent ICI (11, 12). Ocular immune-related side effects occur in approximately 1% of patients and most commonly manifest as dry eyes and inflammatory uveitis. ICI-induced cranial nerve (CN) palsies are seen very infrequently, with only 3 cases reported between 1990 and 2017 in the literature (13). In this case report we describe a 66-year-old female presenting with an isolated CN VI palsy, following recent commencement of ipilimumab and nivolumab immunotherapy for unresectable malignant melanoma.

## 2 Case description

A 66-year-old lady was referred to Dermatology by her general practitioner following new pain and change in colour of a 3mm mole in her upper left arm. The lesion was not ulcerated. No other skin lesions, lymph nodes or sites of metastatic disease were identified on examination. The patient had a background of hypertension, hiatus hernia, knee osteoarthritis with recent right-sided total knee replacement, previous cholecystectomy, previous breast fibroadenoma and allergic sinusitis. She reports multiple drug intolerances including Amitriptyline, Gabapentin, Erythromycin, Co-trimoxazole and an allergy to Amoxicillin. She also reported significant previous sun exposure, having lived in the Far-East for ten years. The skin lesion was subsequently excised by Dermatology in September 2021, with histology revealing a 3x2mm (pT1bNxMx), V600E *BRAF* mutant, superficial spreading malignant melanoma of the left bicep, with a Breslow thickness of 0.95mm and a mitotic rate of 2/mm^2^. In light of her positive histology, she was subsequent referred to the local Plastic Surgery service for a wide local excision, alongside sentinel lymph node biopsy. This procedure was performed in January 2022 without complication. Histology revealed no evidence of melanoma in the scar or the excised lymph node and the patient entered routine follow-up.

Just 6 months later, the patient reported a left-sided neck lump superior to the clavicular midline. Ultrasound identified a malignant looking, 2.2cm supraclavicular lymph node that could not be biopsied due to proximity to surrounding vasculature. Cross-sectional imaging also identified left axillary/retro-pectoral metastases, but no distant metastases. Discussion at the melanoma multi-disciplinary team meeting concluded that these findings were consistent with relapsed Stage III melanoma, and that surgery would be unlikely to achieve a curative clearance.

Resultantly, the patient was review by oncology and consented to ipilimumab 3mg/kg and nivolumab 1mg/kg for the treatment of unresectable Stage III disease. Prior to starting treatment, the patient self-reported a concerning rapid increase in the size of her palpable nodal disease. Seven days after receiving her first treatment cycle, she developed a CTCAE Grade 3 pruritic, papular rash over her chest, back, legs and groin. Considering the likely diagnosis of immune-mediated dermatitis, she was initially treated with betamethasone cream and regular chlorphenamine, with no improvement of symptoms. Following Dermatology review, she commenced topical clobetasol and cycle 2 was deferred, with subsequent improvement to Grade 1 dermatitis. Routine blood tests at this time also demonstrated Grade 1, asymptomatic hyperthyroidism (TSH 0.03, free T4 56) for which she did not require pharmacological intervention.

Three weeks post-cycle 1, the patient was admitted to her local hospital for triage of a new thunderclap headache, photophobia and painless diplopia. A CT head and venogram did not yield any abnormalities and she declined a lumbar puncture to investigate this further prior to discharge. At subsequent review in the oncology clinic, she was noted to have reduced movement of her left eye in the context of no trauma and no fatiguability. An urgent ophthalmology review was organised and the patient was diagnosed with complete left-sided CN VI palsy and bilateral Grade 2 anterior uveitis. She was given an eye patch and started on steroid eye drops by the ophthalmology team. Given the likely diagnosis of a neurological irAE, high dose oral prednisolone (0.5mg/kg/day) was also started by the acute oncology service. An MRI head and orbits scan was also performed at this point, showing no intra-cranial or intra-orbital metastatic deposits, leptomeningeal disease, or structural causes to explain the CN VI palsy. The MRI obits also identified no radiological diagnoses of myositis of the extra-ocular muscles. After 6 days of high dose oral prednisolone, she was able to abduct her left eye slightly past midline and had complete resolution of her headaches. Further treatment with ipilimumab/nivolumab was held and she was placed on a weaning dose regimen of oral prednisolone of 10mg every 5 days. She reported full compliance to prednisolone and had no side effects from steroid use. She was reviewed by ophthalmology after 30 days of combined steroid therapy with a significant improvement of her CN VI palsy and resolution of uveitis. 10 weeks after initiation of immunotherapy, she developed Grade 2 hypothyroidism consistent with the natural disease course of immune-related thyroiditis (14). She also subsequently developed vitiligo on her arms and legs 6 months post-commencement of immunotherapy.

**Figure 1:**
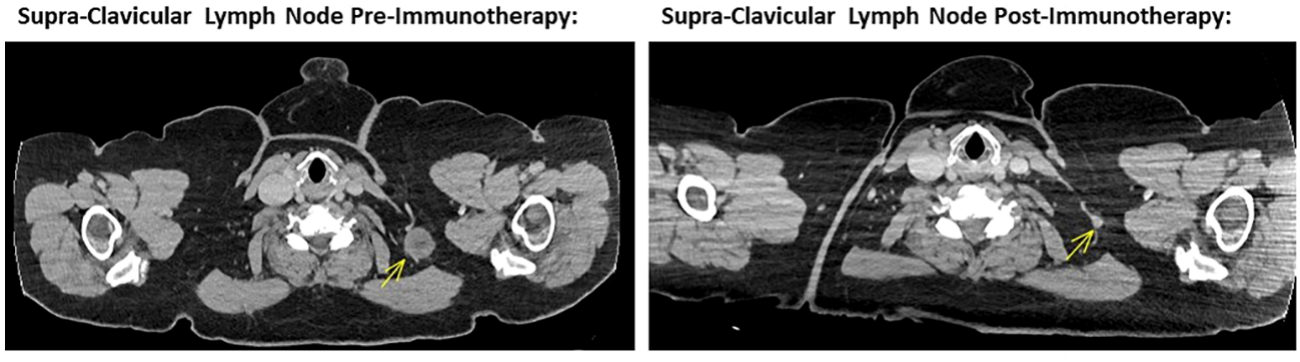
Left image showing cross sectional CT imaging of the head, chest, abdomen and pelvis in November 2022, identifying a 24mm left supra-CLAVICULAR soft tissue lesion compatible with a melanoma metastasis. Right image showing cross sectional CT imaging of the head, chest, abdomen and pelvis in October 2023, noting a tiny left supra-clavicular lymph node measuring 6 mm in short axis. This is in keeping with a marked response to ipilimumab and nivolumab combination immunotherapy.

**Figure 2:**
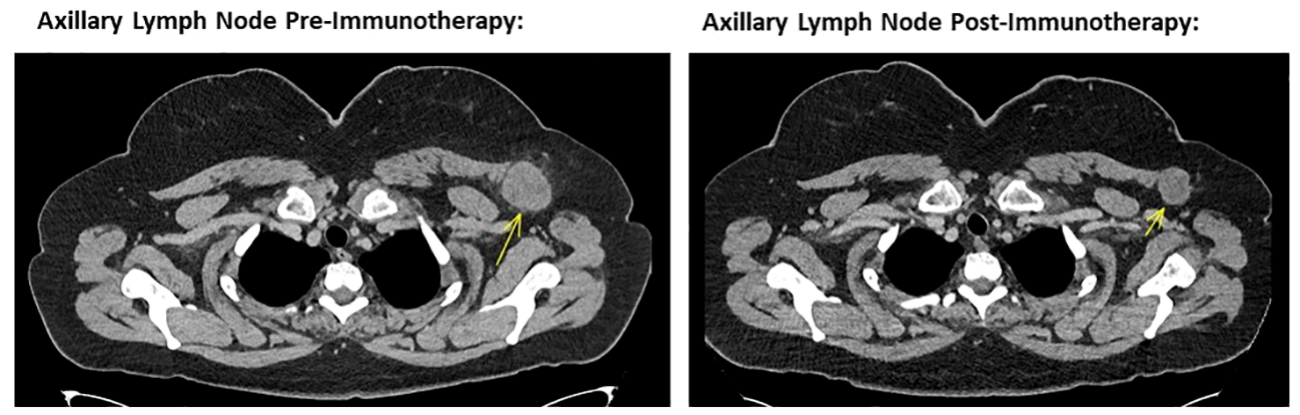
Left image showing cross sectional CT imaging of the head, chest, abdomen and pelvis in November 2022, identifying a 40mm left axillary lesion compatible with a melanoma metastasis. Right image showing cross sectional CT imaging of the head, chest, abdomen and pelvis from October 2023, noting the same left axillary lesion measuring 28 mm in short axis. This is consistent with a marked response to ipilimumab and nivolumab combination immunotherapy.

The patient self-reported a considerable and ongoing clinical response, with a reduction in the size of her palpable disease contemporaneous with the immunotherapy-related toxicities she experienced. At clinical review 12 weeks after starting treatment, her left sided nodal disease was impalpable with a restaging CT scan showing good partial response in the axillary and supraclavicular disease (**Figures 1**, **2**) and no new sites of disease identified. Considering the marked toxicities experienced, further immunotherapy was suspended. The patient currently remains on surveillance, with a 6 and 9-month restaging CT showing an excellent, continued partial response.

## 3 Timeline of events

**Figure 3:**
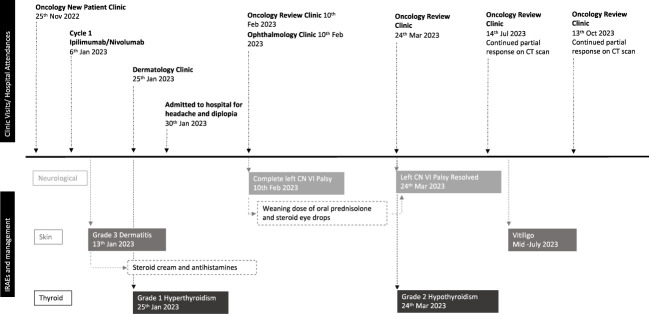
Timeline of events relating to treatment and toxicity.

As depicted in **Figure 3**.

## 4 Discussion

Immune-mediated neurotoxicity varies considerably in both severity and site. In cases of melanoma and treatment with ICI, significant neurological irAEs are reported to occur in approximately 1% of patients. Previously reported neurological toxicities post-immunotherapy include peripheral neuropathies, bulbar palsies, encephalitis, Guillian-Barre syndrome and aseptic meningitis (11). The literature also tells us that neurological irAEs are more common with CTLA-4 & PD-1/PD-L1 combination therapies (12), and incidence is typically dose-dependent (8). Interestingly, no patients enrolled in CheckMate 067 experienced neurological adverse events. This is the first reported case study of an isolated CN VI palsy following immunotherapy in the UK and, to our knowledge, the first reported case in a patient treated with combination ipilimumab and nivolumab; it has previously been reported elsewhere following the use of pembrolizumab in a patient with stage IV melanoma (15). Other cranial nerve palsies, including combined CN VI and VII palsy have also previously been reported with single agent nivolumab (13, 16).

Palsies of CN VI result in impaired innervation of the ipsilateral lateral rectus (LR) muscle, responsible for abduction of the eye. The nerve runs a long anatomical path from its origin and the site of lesion within the nerve often predicts the underlying cause of dysfunction (17). Possible differentials for causes of isolated CN VI palsy include ischaemia, neoplasm, aneurysms, inflammation and infection (17). In this patient’s case, MRI imaging ruled out aneurysms and metastatic deposits; both bloods and imaging were also atypical for an infective cause. Mindful of the patient’s background of hypertension, we note that her blood pressure was well-controlled on amlodipine, and she had no other clinical evidence of atherosclerosis or cardiovascular disease. Furthermore, at presentation with diplopia the patient’s serum blood glucose was 5.1mmol/L and lipid levels were unremarkable. Supporting ipilimumab and nivolumab as a cause of the patient’s CN IV palsy, this toxicity occurred just weeks after commencing treatment. Published literature highlights that most neurological irAEs will occur within 3 months of ICI commencement, in those patients who will experience them (10). The patient’s rapid response to steroids also supports this conclusion. In the aforementioned case study of an isolated CN VI palsy after single agent Pembrolizumab, the patient’s symptoms also improved rapidly on commencement of steroids, with complete resolution of neurological deficit by day 43 (15).

Another interesting facet of this case was the development of multiple different irAEs. Those experiencing one ICI toxicity are known to be more likely to experience at least one other (18). Multiple irAEs are more common in patients treated with combination ipilimumab and nivolumab therapy (36%) than with single agent anti-PD1 therapy (17-23%) (19, 20). This highlights the need to perform a comprehensive assessment of patients presenting with irAEs to detect additional toxicity. Additionally, despite the suspension of further ICI, these patients remain at risk of subsequent IRAEs, as seen in our case, and follow-up should be tailored accordingly to aid detection of these. In keeping with this lady’s tumour response to date, the occurrence of multiple irAEs is associated with more favourable outcomes in several tumour types, including melanoma (10). It has been hypothesized that patients experiencing isolated IRAEs may be predisposed to that specific irAE, whereas the occurrence of multiple IRAEs may represent enhanced systemic immune activation secondary to ICI (21, 22). An important area for further study would be to distinguish if irAEs occur more frequently or to a more severe extent in patients who are innately more susceptible to allergies.

## 5 Conclusion

In this case, we present the first reported case of immunotherapy-induced, isolated, CN VI palsy in the UK; it is one of few reported cases worldwide. Though ICI continue to revolutionise oncological care for many patients, such cases must remind us of the unpredictability of their toxicities (sometimes with fatal consequences) and the importance of prompt, comprehensive assessment and intervention.

## 6 Patient perspective

Overall, the patient felt that she was appropriately counselled during the consenting process on the potential immunotherapy-related side effects she could experience while on treatment. She expressed that she had made an independent and well-informed decision to proceed with combination ipilimumab and nivolumab. While her multiple ocular, skin and thyroid toxicities were an inconvenience, she was pleased that they could all be managed pharmacologically if necessary and have mostly resolved with time. She did not feel systemically unwell while on treatment, which was important to her. She feels that the durable treatment benefit, in the form of continued partial response at present, outweighs the toxicities she experienced and does not regret receiving immunotherapy.

## Data availability statement

The original contributions presented in the study are included in the article/supplementary material. Further inquiries can be directed to the corresponding author.

## Ethics statement

Written informed consent was obtained from the individual(s) for the publication of any potentially identifiable images or data included in this article.

## Author contributions

SL: Writing – original draft, Writing – review & editing. KE-S: Writing – original draft, Writing – review & editing. EB: Writing – review & editing. AC: Writing – review & editing. SM: Writing – review & editing. MS: Conceptualization, Supervision, Writing – review & editing.
